# Evaluating the Impact of an App-Delivered Mindfulness Meditation Program to Reduce Stress and Anxiety During Pregnancy: Pilot Longitudinal Study

**DOI:** 10.2196/53933

**Published:** 2023-12-25

**Authors:** Donna Balsam, Dawn T Bounds, Amir M Rahmani, Adeline Nyamathi

**Affiliations:** 1 School of Nursing San Diego State University San Diego, CA United States; 2 Sue & Bill Gross School of Nursing University of California, Irvine Irvine, CA United States

**Keywords:** mindfulness app, pregnancy, pregnant, maternal, obstetric, obstetrics, stress, anxiety, heart rate variability, mindfulness, mHealth, mobile health, app, apps, applications, mental health, meditation, mind-body, complementary, alternative, heart rate, sleep, mobile phone

## Abstract

**Background:**

Stress and anxiety during pregnancy are extremely prevalent and are associated with numerous poor outcomes, among the most serious of which are increased rates of preterm birth and low birth weight infants. Research supports that while in-person mindfulness training is effective in reducing pregnancy stress and anxiety, there are barriers limiting accessibility.

**Objective:**

The aim of this paper is to determine if mindfulness meditation training with the Headspace app is effective for stress and anxiety reduction during pregnancy.

**Methods:**

A longitudinal, single-arm trial was implemented with 20 pregnant women who were instructed to practice meditation via the Headspace app twice per day during the month-long trial. Validated scales were used to measure participant’s levels of stress and anxiety pre- and postintervention. Physiological measures reflective of stress (heart rate variability and sleep) were collected via the Oura Ring.

**Results:**

Statistically significant reductions were found in self-reported levels of stress (*P*=.005), anxiety (*P*=.01), and pregnancy anxiety (*P*<.0001). Hierarchical linear modeling revealed a statistically significant reduction in the physiological data reflective of stress in 1 of 6 heart rate variability metrics, the low-frequency power band, which decreased by 13% (*P*=.006). A total of 65% of study participants (n=13) reported their sleep improved during the trial, and 95% (n=19) stated that learning mindfulness helped with other aspects of their lives. Participant retention was 100%, with 65% of participants (n=13) completing about two-thirds of the intervention, and 50% of participants (n=10) completing ≥95%.

**Conclusions:**

This study found evidence to support the Headspace app as an effective intervention to aid in stress and anxiety reduction during pregnancy.

## Introduction

### Overview

Stress and anxiety during pregnancy are extremely prevalent, where an estimated 58% of people who are pregnant experience prenatal stress [1], and 25% experience anxiety [2]. Stress and anxiety during pregnancy are associated with numerous poor outcomes, among the most serious are increased rates of preterm birth [3] and low birth weight infants [4]. According to the World Health Organization (WHO), preterm birth is the leading cause of infant morbidity and mortality in children worldwide [5], with an estimated 15 million preterm infants born annually [6]. In the United States, approximately 11% of infants are born preterm [7], leading to societal costs of US $26 billion each year [8].

Mind-body practices like yoga and mindfulness meditation are becoming increasingly popular to manage stress and anxiety. Mindfulness is the awareness that arises through paying attention, on purpose, in the present moment, nonjudgmentally [9]. There is evidence that mindfulness meditation training can have a beneficial impact on reducing perinatal stress [10] and anxiety [11]. However, in-person instruction has barriers including accessibility, availability, cost, and time.

Internet mindfulness-based interventions (iMBIs), including computer and app-based resources, provide a convenient alternative to traditional mindfulness classes. Studies examining the impact of iMBIs during pregnancy found a reduction in stress [12,13] and anxiety [14,15]. However, limitations included high attrition, poor adherence, and lack of objective or physiological measures of stress, like heart rate variability (HRV) and sleep. Research supports that HRV is an objective, reliable measure of stress [16]. Sleep is also reflective of stress, where improved sleep reduces stress [17]. None of the reviewed studies used top-rated iMBIs like the Headspace app. Headspace is among the highest-scoring mindfulness apps based on the Mobile Application Rating System [18]. The purpose of this study was to determine if a more accessible form of mindfulness meditation training, specifically Headspace, can help reduce stress and anxiety during pregnancy.

### Theoretical Framework

The theoretical framework for this study is based on the polyvagal theory [19], which postulates that the vagus nerve has two branches that regulate different physiological states: the dorsal vagal complex, associated with immobilization behaviors (rest or digest and shutdown or freeze), and the ventral vagal complex, associated with social engagement, calm, and safety [19]. Relaxation practices like meditation activate the ventral vagal complex, reducing stress [20], and improving HRV [21]. Various HRV metrics, which are reflective of vagus nerve activity, can be extracted. For example, the HRV metric the root-mean-square of successive differences between adjacent normal heartbeats (RMSSD) is mediated by the vagus nerve [22], with higher values indicating relaxation and lower mental stress [23]. The HRV metric low-frequency (LF), reflects a mix of the sympathetic and vagal influences on HRV [24], and lowers with relaxation practices, like mindfulness [25,26].

### HRV Changes in Pregnancy

Most HRV metrics decrease throughout pregnancy [27,28]. A recent study, which evaluated HRV changes during pregnancy against age-matched nonpregnant controls, found that there was reduced HRV in the pregnant groups for all trimesters [29]. A more recent systematic review evaluating 8 research studies measuring HRV trends across gestation in healthy pregnant women found further evidence that HRV decreases across gestation for all HRV metrics but 2. The LF and the LF per high-frequency (HF) ratio showed an ascending trend from early to late pregnancy [28]. Despite the variations in HRV observed during pregnancy as compared to the nonpregnant population, several studies have used HRV as a physiological measure of stress during pregnancy [26,30-32].

## Methods

### Participants

**Figure 1:**
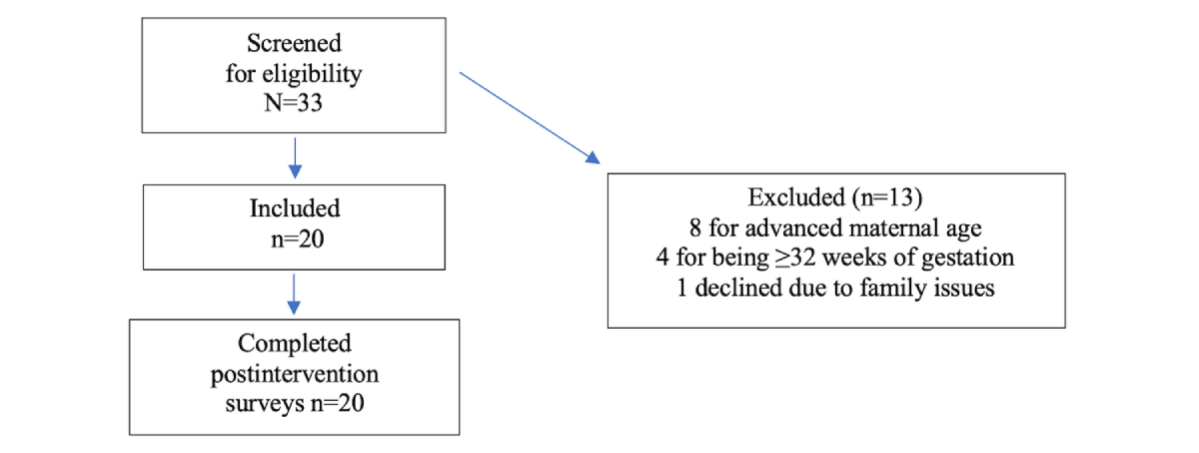
CONSORT diagram. CONSORT: Consolidated Standards of Reporting Trials.

The study sample consisted of 20 study participants to achieve 80% power with a moderate effect size (0.35) and α of .05. The inclusion criteria were (1) pregnant people residing in San Diego County; (2) age 18-35 years; (3) between 10 and 32 weeks gestation; (4) able to read and understand English; and (5) access to a smartphone, Wi-Fi, email, and able to download apps. Exclusion criteria were (1) people who regularly (≥3× per week) engaged in other mindfulness practices like yoga, (2) concurrent enrollment in a mindfulness meditation class, (3) hearing impairment, (4) cognitive impairment, (5) chronic health or pregnancy-related medical condition causing “high risk” pregnancy, (6) current psychotherapy, (7) current psychoactive medications, and (8) severe depression or anxiety. A total of 33 people were screened for eligibility, with 8 excluded for advanced maternal age, and 4 were excluded because they had passed 32 weeks of gestation (Figure 1).

### Ethical Considerations

After the institutional review board approval from the University of California, Irvine (HS# 2021-6664), recruitment flyers were posted at maternity clinics, describing the study, and providing contact information. Once contacted, potential participants were sent a recruitment email containing further study details and instructions to contact the investigator if they met eligibility criteria. Next, a phone conversation was scheduled to further assess eligibility, provide details, and answer questions. During the call, potential participants were asked to give their verbal consent for the investigator to further assess their eligibility, and screen them for severe anxiety and depression. Participants were informed that all records would be kept confidential and stored in REDCap (Research Electronic Data Capture; Vanderbilt University), a Health Insurance Portability and Accountability Act of 1996 (HIPAA)–compliant web-based app for managing clinical research data.

### Design and Procedure

Potential participants were screened for severe depression using the Edinburgh Postnatal Depression Scale (EPDS) [33] and severe anxiety using the Generalized Anxiety Disorder Scale (GAD-7) [34]. EDPS scores of ≥19 and GAD-7 scores of ≥15 rendered potential participants ineligible. Next, a meeting was arranged for consent, completion of baseline measures, and assistance with study apps and materials (Multimedia Appendix 1). Participants were loaned an Oura Ring to wear nightly during the study period. Participants wore the ring for 4 days (2 workdays and 2 days off) prior to starting the intervention to get a baseline HRV reflective of their stress levels. The researcher monitored the Oura Ring server throughout the experiment to assess participant compliance with wearing the Oura ring, and that data were recorded. Surveys and measures of stress and anxiety were completed at baseline and study completion, and measures for anxiety were additionally collected at the study midpoint (Multimedia Appendix 2).

### Incentive

As a study incentive, participants each received a 6-month subscription to the Headspace app, as well as US $50. Once mid-study surveys were completed, participants received a US $15 gift card incentive by email. At study completion, once the participants finished the final study measures, they were contacted by the investigator to arrange a meeting time and place to return their Oura Ring and charger and receive their remaining financial incentive (US $35 gift card).

### Materials: Headspace

Participants were instructed to practice meditation with Headspace twice daily during the month-long trial, starting with “Basics,” followed by “Pregnancy.” “Basics” contained 30 meditations, between 3 and 20 minutes long, introducing the essentials of mindfulness meditation. Next, participants were instructed to complete 30 meditations in the “Pregnancy” course, between 10 and 20 minutes long, which focused on the development of favorable conditions for pregnancy and birth. The total intervention was 60 meditations for a range of 530-1050 minutes.

### Measures

#### Screening Measures

##### Screening Tool

A screening tool was used to assess eligibility via the inclusion and exclusion criteria.

##### Severe Depression

Potential study participants were screened for severe depression using the EPDS [33]. The EDPS has been validated against both the Diagnostic and Statistical Manual of Mental Disorders, Fifth Edition (DSM-5) and the International Classification of Diseases, Tenth Revision (ICD-10), with high sensitivity and specificity [35]. Scores of this 10-item scale range from 0 to 30, with higher scores indicating the presence of depressive symptoms.

##### Severe Anxiety

Potential study participants were screened for severe anxiety using the GAD-7 [34]. The GAD-7 assesses the most prominent diagnostic features from the DSM-5 for generalized anxiety disorder [36]. The GAD-7 asks respondents to rate how often they experienced the survey anxiety symptoms within the last 2 weeks. Scores range from 0 to 21, and scores of 5, 10, and 15 are the respective cut-off points for mild, moderate, and severe anxiety [34]. The GAD-7 has demonstrated good reliability (Cronbach α=.89) and validity in the pregnant population [37]. This measure was additionally used as an anxiety assessment at baseline and study completion.

#### Other Baseline Measure: Social Support

Social support was assessed using the Multidimensional Scale of Perceived Social Support (MSPSS) [38]. The MSPSS consists of 12 questions using a 7-item Likert scale for participant responses, ranging from “very strongly disagree” (1) to “very strongly agree” (7), with higher numbers indicating greater social support. The MSPSS has shown good-to-excellent internal reliability with a Cronbach α of .81-.98, and test-retest reliability of 0.92-0.94 [38].

#### Physiologic Measures and Dependent Variables

##### Heart Rate Variability

HRV data were collected by the Oura Ring, which wirelessly syncs data to the Oura app and server. The Oura Ring uses photoplethysmography for HRV monitoring, a commonly used signal in wearable technology designed for stress measurement. Oura Ring measurements of HRV are highly accurate [39]. Various HRV metrics were extracted and analyzed, with each metric correlating with a physiological representation of stress, depending on whether their values are low or high. To evaluate the impact of an intervention on these metrics, we can observe if the values increase or decrease. As the most accurate HRV assessments are recorded with the participant at rest in the supine position [40], HRV data were collected during participant sleep hours.

##### Sleep

Sleep data were collected by the Oura Ring, which analyzes sleep by measuring the following: resting heart rate (HR), body temperature, time spent in specific sleep stages (including light, deep, and rapid eye movement), and movement [41] via actigraphy, a validated method of measuring sleep via an accelerometer [42]. Oura’s algorithms combine the measurements, yielding a sleep score ranging 0-100, with higher scores indicating better sleep. Oura Ring measurements of sleep are highly accurate [43].

#### Self-Report Measures

##### General Anxiety and Pregnancy Anxiety and Dependent Variables

To evaluate anxiety, the GAD-7 (see above) and the Pregnancy-Related Anxiety Scale (PRAS) [44] were used. PRAS is a measure specific to pregnancy anxiety (eg, worries about health during pregnancy and childbirth). This 10-item instrument used a Likert scale, where participants answered questions with options ranging from “never or not at all” (1) to “a lot of the time or very much” (4). Scores range from 0 to 30, with higher scores indicating greater anxiety. This scale has good internal reliability, with a Cronbach α of .78 [44].

##### Stress, Dependent Variable

Stress was measured using the Perceived Stress Scale (PSS) [45], a questionnaire that was designed to measure how uncontrollable and overloaded participants perceive their lives to be [45]. The PSS-10 is a 10-item, 5-point Likert scale assessment, which asks participants questions based on their thoughts or feelings over the previous month. Scores range from 0 to 40, with higher scores indicating higher stress. The PSS has good psychometric qualities [45,46], with good internal consistency and a Cronbach α of .89 [46].

##### Investigator-Developed Items

Investigator-developed items were collected at baseline and study completion to assess potentially confounding lifestyle information (medications, substance use, and exercise). An example Likert-style question used in the exit survey was “My exercise level increased during this trial” with answers ranging from “strongly disagree” to “strongly agree,” and an open-ended question asking how learning and practicing mindfulness meditation helped with other aspects of their life.

### Data Analysis

HRV and sleep data were obtained from the Oura dashboard and analyzed. To extract HRV features, we used the interbeat interval (IBI) data. Oura calculates HR and IBI using the optical photoplethysmography sensor embedded in the Oura Ring. Time-domain variables (HR, RMSSD, and SD of the IBI of normal sinus heartbeats, with “normal” referring to the removal of artifact [SDNN]) were extracted through statistical analysis, and frequency domain variables (LF, HF power bands, and LF per HF ratio) were extracted and analyzed by fast Fourier transform and power spectral density algorithms [16,22]. To assess potential physiological changes reflective of reduced stress (HRV and sleep changes over time), hierarchical linear modeling (HLM) was used.

To evaluate subjective changes in stress, anxiety, and pregnancy anxiety, the self-report measures (PSS, GAD-7, and PRAS) were assessed at baseline and study completion. Paired 1-tailed *t* tests compared the before and after observations on the same subjects. Subjective sleep was assessed with a Likert scale prompt on the postintervention questionnaire: “My sleep improved during this trial,” with options ranging from “strongly disagree” to “strongly agree.”

## Results

### Participants

Most study participants were White (n=16, 69.6%), educated (n=18, 90% completed college), with high social support (n=18, 90%), married (n=15, 75%), working full-time (n=14, 70%), and an average age of 29.45 years (Table 1). Data on exercise were collected as an increase in exercise level during the trial could have been a potential confounder. At baseline, 50% (n=10) of the women reported exercising regularly. Use of certain medications, alcohol, tobacco, recreational drugs, and excessive caffeine can impact HRV [47], so baseline data on participant usage were collected. All participants denied taking medications that are known to impact HRV, and 100% (n=20) of the participants denied use of alcohol, recreational drugs, tobacco, and vaping. All participants indicated they consumed low-to-no caffeine (≤1-2 cups per day). At baseline, 60% (n=12) of participants reported regular stress-management activities (eg, prayer and massage). Participants’ baseline stress and anxiety levels were low-to-moderate. Baseline stress (PSS) levels were as follows: 50% (n=10) had low, 45% (n=9) had moderate, and 5% (n=1) had high stress. Baseline general anxiety (GAD-7) levels were as follows: 65% (n=13) had mild, 25% (n=5) had moderate, and 10% (n=2) had severe general anxiety. Baseline pregnancy anxiety (PRAS) was interpreted as mild to moderate (mean score 10.84, SD 5.2).

Parity and gestation trimester can impact HRV [27]. Most study participants were primiparous (n=12), while the remainder were multiparous. At the start of the study, 30% of participants (n=6) were in their first trimester, 55% (n=11) were in their second trimester, and 15% (n=3) were in their third trimester. During the month-long trial, all participants stayed within a week range of their original trimester, except for 3 who changed, 1 from first to second and 2 from their second to their third trimester. At study completion, 25% (n=5) were in their first trimester, 50% (n=10) were in their second trimester, and 25% (n=5) were in their third trimester (Table 1).

**Table 1:** Sample baseline characteristics.

Characteristics		Values
Age, mean (SD)		29.45 (3.27)
**Race or Ethnicity (including dual ethnicity identity), n (%)**		
	White or European American	16 (69.6)
	Hispanic or Latino	4 (17.4)
	Asian or Pacific Islander	2 (8.7)
	Black or African American	0 (0.0)
	Other	1 (4.3)
**Parity, n (%)**		
	Primiparous	12 (60)
	Multiparous	8 (40)
**Trimester of gestation, n (%)**		
	First trimester	6 (30)
	Second trimester	11 (55)
	Third trimester	3 (15)
**Education, n (%)**		
	Completed master’s degree or higher	6 (30)
	Completed bachelor’s degree	8 (40)
	Completed associate degree	4 (20)
	Completed high school or equivalent	2 (10)
**Relationship type, n (%)**		
	Married	15 (75)
	Cohabitating	3 (15)
	Single	2 (10)
**Employment status, n (%)**		
	Full-time	14 (70)
	Part-time	3 (15)
	Unemployed	3 (15)
**Exercise, n (%)**		
	Regular exercise	10 (50)
	No regular exercise	10 (50)
	Denied alcohol, recreational drugs, or vaping	20 (100)
**Caffeine, n (%)**		
	Denied caffeine use	7 (35)
	Drank 1-2 cups of caffeinated drinks per day	13 (65)
**Regular stress-management activities, n (%)**		
	Yes	12 (60)
	No	8 (40)
**Social support, mean (SD)**		6.14 (0.73)
	High social support	18 (90)
	Moderate social support	2 (10)
	Low social support	0 (0)

### HRV

The HLM analysis of the physiological data showed changes in HRV reflective of reduced stress (Table 2). A total of 1 of 6 HRV metrics, LF, changed in the direction of reduced stress, lowering by 13% (*P*=.006). The LF per HF ratio changed in the direction of reduced stress, decreasing by 2% and trending toward significance (*P*=.09). The statistically significant results in 2 additional HRV metrics, HR and RMSSD, moved in the opposite direction of what was hypothesized, with minimum or resting HR increasing by 2.3% (*P*=.007), and RMSSD decreasing by 9% (*P*=.007). SDNN (*P*=.09) and HF (*P*=.07) both decreased, moving opposite of the hypothesized direction.

**Table 2:** Results of physiological stress outcome measures.

Monitoring parameter or metric			Expected direction of change indicating reduced stress [26]		% change in 4 weeks		*P* value
**HRV^a^ metric**							
	Min HR^b^ (resting)	↓		Increased by 2.3		.007	
	RMSSD^c^	↑		Decreased by 9		.007	
	SDNN^d^	↑		Decreased by 7		.09	
	HF^e^	↑		Decreased by 11		.07	
	LF^f^	↓		Decreased by 13		.006	
	LF per HF ratio	↓		Decreased by 2		.09	
**Sleep metric**							
	Sleep score	↑		Increased by 2		.09	

^a^HRV: heart rate variability.

^b^HR: heart rate.

^c^RMSSD: the root mean square of successive differences between adjacent normal heartbeats.

^d^SDNN: SD of the IBI of normal sinus heartbeats, with “normal” referring to the removal of artifact.

^e^HF: high-frequency.

^f^LF: low-frequency.

### Stress

Paired *t* test analysis yielded statistically significant reductions in stress (*P*=.005) from baseline to postintervention (Table 3).

**Table 3:** Pre- and postintervention mean comparisons for self-report variable.

Measure	Pre, mean (SD)	Post, mean (SD)	*P* value
PSS^a^	14.4 (5.83)	10.25 (5.4)	.005
GAD-7^b^	4.65 (3.25)	2.6 (2.93)	.01
PRAS^c^	10.84 (5.2)	5.9 (3.99)	.0001

^a^PSS: Perceived Stress Scale.

^b^GAD-7: General Anxiety Disorder Scale.

^c^PRAS: Pregnancy-related Anxiety Scale.

### General Anxiety and Pregnancy Anxiety

Paired *t* test analysis yielded statistically significant reductions in anxiety (*P*=.01) and pregnancy anxiety (*P*=.0001) from baseline to postintervention (Table 3).

### Sleep

HLM analysis of sleep indicated that sleep score increased by 2%, trending toward significance (*P*=.09; Table 2), which reflects decreased stress. On the exit survey, 13 (65%) participants reported their improved sleep during the trial.

### Other

Regarding potential confounders, none of the study participants were on medications or used substances that may have impacted their HRV. A total of 9 study participants indicated that their level of exercise increased during the study period. Of the 13 participants who reported improved sleep during the trial, 6 reported increased exercise.

An exit survey question asked, “Did learning/practicing mindfulness meditation help with other aspects of your life?” The overwhelming majority, 19 participants, answered “yes.” A total of 17 participants provided narrative details, and a quasi-qualitative thematic evaluation identified that learning and practicing mindfulness meditation helped study participants with the following: improved patience, improved perspective, improved focus, improved conflict management, reduced emotional reactivity, and a greater focus on self-care. All participants completed the postintervention assessments, received their financial incentive, and returned the Oura Rings and chargers, indicating a 100% retention rate, with no attrition.

## Discussion

### Principal Findings

This study found evidence to support that the Headspace app is an impactful mindfulness meditation intervention to aid in stress and anxiety reduction for the pregnant population. Study results indicated that LF, 1 of 6 HRV parameters evaluated in this study, decreased significantly by 13% (*P*=.006). LF reflects both sympathetic nervous system (SNS) and parasympathetic activity, with the SNS and baroreceptor activity playing a large role in generating this frequency [22]. A recent systematic review which included 8 studies analyzing HRV among healthy pregnant women found that LF showed an ascending trend from early to late pregnancy, indicating that an increase in sympathetic activity is common in pregnancy [28]. As lower levels of power in the LF band indicate relaxation and low mental stress [48], the reduced levels of LF in this trial support the efficacy of the intervention for stress reduction among study participants. Another HRV metric, LF per HF ratio, decreased by 2%, trending toward significance (*P*=.09). The LF per HF ratio is a commonly used index of sympatho-vagal balance and generally increases with stress [23,48]. Therefore, the decreased value suggests reduced mental stress for study participants.

In the literature using HRV metrics as outcome variables, when one of the metrics changed in the direction expected for a stress-reducing intervention, the findings were reported as evidence that the intervention was effective [30,31]. Therefore, the HRV results in this study follow current reporting trends.

In this trial, the HLM analysis indicated that the participants’ HR measure changed in the opposite direction of what was hypothesized, increasing by 2.3% (*P*=.007). For the general population, it is expected that a stress-reducing intervention would cause a decrease in HR. However, during pregnancy, there is an expected increase in HR. A recent meta-analysis evaluating trends in HR among 8317 pregnant women found that on average, the HR during pregnancy increased by 10% (7.6 beats per min) [49]. As the increase in HR for this pilot study population was 2.3% (*P*=.007), it is possible that the intervention impacted the participant’s HR, potentially causing a smaller increase than would have been expected without a stress-reducing intervention. It could be that the impact was less pronounced due to the normal physiological pregnancy changes impacting HR.

The remaining HRV results in this pilot study were consistent with current research findings regarding the expected direction of HRV metrics during pregnancy. A recent study examining HRV trends across pregnancy compared to unpregnant matched controls found that SDNN, RMSSD, LF, and HF metrics decreased significantly during the second trimester [27]. Half of the women in this pilot study were in their second trimester, which could explain the decreased levels of these metrics observed in this experiment. Furthermore, it has been found that HRV reduction in pregnancy is more marked for primiparous versus multiparous women [29]. A total of 12 (60%) of the women in this pilot study were primiparous, which may have contributed to the study findings.

A total of 13 (65%) participants reported an improvement in their sleep during the trial, and the physiological data analysis found that sleep improved by 2%, trending toward statistical significance (*P*=.09). Exercise may have been a confounder, as exercise is associated with improved sleep and reduced stress [10] and anxiety [50]. Given that 9 (45%) study participants reported increased exercise during the trial, there is a question as to whether the improvement in their stress, anxiety, and sleep could have been related to exercise. A narrative review which included 13 studies evaluating mindfulness interventions during pregnancy noted that none of the studies evaluated sleep and asserted that it is imperative to evaluate sleep in this population [51]. This study contributes to the literature by including sleep as an outcome variable and by providing preliminary data that practicing mindfulness meditation with the Headspace intervention may improve sleep during pregnancy.

Participants were asked to complete 60 meditations, for a total range of 530-1050 minutes of meditation. A total of 13 of 20 participants completed ≥65% of the intervention, with 10 of those participants completing ≥95% of the intervention. This supports the acceptability and feasibility of Headspace as a mindfulness meditation intervention for this population.

### Conclusion, Study Strengths, and Limitations

The use of technology via the Oura Ring and Headspace enabled the collection of objective data (HRV, sleep, and intervention-time usage), which contributed to validity. Using the Oura Ring to collect HRV data yielded approximately 3000 data points per individual, reducing the standard error in the results, and contributing to the overall study strength. The study design included best practices related to HRV collection, and the inclusion of recommended HRV variables [52]. That the study participants did not partake of potentially confounding substances (eg, alcohol and tobacco) was a strength, as was the collection of data that were potential confounders. There was a 100% participant retention rate, and adherence to the intervention was very good, providing support for the feasibility of this study design, and the usability and feasibility of the intervention.

The study may have been underpowered due to the sample size and potential lack of time engaging with the intervention to achieve significant findings in some HRV metrics. The sample population was homogenous, consisting of predominantly White, educated, women of low to moderate stress, and who self-selected to participate, which limits the generalizability of study results. Future research needs to be done with a more diverse study sample. Participants may have been influenced by the recommendations provided by the Oura Ring app. Subjective sleep was not evaluated with a validated scale and the qualitative analysis may have been biased as the investigator was the sole interpreter. The GAD-7 assesses anxiety within the past 2 weeks. Recommendations for future research include a stronger study design, such as a randomized controlled trial (RCT) with a mixed methods component; for the RCT to have controls that are matched by factors that impact HRV (parity, gestational age, and maternal age); inclusion of a validated self-report sleep measure; objective collection of exercise data; and having a large enough sample size to independently analyze HRV trends per trimester. This study adds to the body of scientific knowledge supporting that mindfulness meditation, and specifically the Headspace app, is an effective iMBI for stress and anxiety reduction among the pregnant population. While future research is necessary, this pilot study showed promising initial results.

## Appendix

Multimedia Appendix 1 Instructions and product information.

Multimedia Appendix 2 Study timeline.

## Abbreviations

DSM-5: Diagnostic and Statistical Manual of Mental Disorders, Fifth Edition
EPDS: Edinburgh Postnatal Depression Scale
GAD-7: Generalized Anxiety Disorder Scale
HF: high-frequency
HIPAA: Health Insurance Portability and Accountability Act of 1996
HLM: hierarchical linear modeling
HR: heart rate
HRV: heart rate variability
IBI: interbeat interval
ICD-10: International Classification of Diseases, Tenth Revision
iMBI: internet mindfulness-based intervention
LF: low-frequency
MSPSS: Multidimensional Scale of Perceived Social Support
PRAS: Pregnancy-Related Anxiety Scale
PSS: Perceived Stress Scale
RCT: randomized controlled trial
REDCap: Research Electronic Data Capture
RMSSD: root mean square of successive differences between adjacent normal heartbeats
SDNN: SD of the IBI of normal sinus heartbeats, with “normal” referring to the removal of artifact
SNS: sympathetic nervous system
WHO: World Health Organization
