# Supplementary material for: Evaluating the Impact of an App-Delivered Mindfulness Meditation Program to Reduce Stress and Anxiety During Pregnancy: Pilot Longitudinal Study
Source: JMIR Pediatr Parent. 2023 Dec 25;6:e53933. doi: 10.2196/53933 (PMC10775027; doi:10.2196/53933)
Supplement: Multimedia Appendix 1 [file pediatrics_v6i1e53933_app1.pdf]

Headspace App:

- Basic directions:
  - While practicing the mindfulness meditation exercises, your focus should be on the guide is teaching you. For example, you should not have it on in the background while cooking or driving.
  - Give yourself the allotted time to do the meditation exercises, which range from 5 to 20 minutes, based on your selection.
  - Consider making a goal to do it at about the same time each day, with a recommendation to do it first thing in the morning. However, the time of day you choose isn't as important as the consistency of doing it every day.
  - Consider setting a daily reminder on your smart phone.
  - Find a space to sit, either on a cushion or chair, as long as you're comfortable.
  - The place should be relatively quiet, free from distractions.
  - You may listen with headphones.
  - Straighten your back to sit comfortably without slouching.
  - Bring your awareness to your breath and follow the prompts of your guide.
- Watch "Getting Started" on the app.
- To set up Reminders on the app, follow these steps:
  - Tap the "Profile" icon on the top left corner of the app
  - Tap on the Settings gear next to your name at top left
  - Select "Notifications" and then "Reminders"
  - Slide the "Remind me" switch to on
  - Slide the "Put it on my calendar" switch to on and tap "Allow" for notifications if you want the reminders added to your calendar
  - Set the time and frequency you want to be notified and your settings will be saved

Oura Ring & Smartphone App:

- The Oura Ring and Smartphone Application (App) collects physiological measures like heart rate variability, heart and respiratory rate, activities, and sleep quality.
- The Oura Ring App can report:
  - Sleep quality and duration: length, quality (REM, Deep, and Light), disruptions in sleep, movement during sleep, awake times, sleep latency, time in bed, sleep efficiency, sleep score.
  - Readiness score: a measure showing how the body responds to and recovers from the demands of daily life; generated using all of the sensor data, physiological signals, sleep and activity patterns that are monitored by the ring.
- <u>For the purpose of this study, the Oura Ring will collect objective data on stress, which is heart rate variability (HRV).</u>
- The app is free to download from both the App Store (IOS) or Google Play Store (Android).
- After the ring is set up with the app, you can open the app each morning to retrieve sleep and recovery data.

- Please open the app every 1-2 days to facilitate the transfer of HRV data to our server.
- For this study, wear the ring at night, during sleep hours
    - Suggestion: put it on at night when brushing your teeth, take it off in the morning when brushing your teeth, and place it in the charger unit. Please store carefully as it is valuable ($300).
- You may wear the ring 24/7 if you choose.
- Ensure sensors are on the palm side of the finger, with the flat part of the ring away from your palm.
- The ring is water (and hand sanitizer) resistant, so it can get wet.
- You may shower with it on, but please remove if engaging in water sport activities like swimming.

Charging

- Battery life is 5-7 days.
- The light on the charger will pulse and turn solid when ring is fully charged.
- When connected to Bluetooth, you can see the ring's battery level by tapping the circle in the top right corner of the Oura app.
- The app will also remind the wearer to charge when battery gets low.
- Charging is quick: takes <1 hour.

For this study:

- The Oura Ring will be worn for the study period, during the hours of sleep.
- Please ensure you sleep with the ring on for 4 nights (2 weekend, 2 weekday) prior to starting the Headspace meditation intervention (this will allow us to obtain a baseline HRV).
- The investigator will give participants an overview of the Oura Ring and Smartphone Application at the baseline visit.
- The investigator will meet with the participants at baseline to distribute the Oura Ring, and one month later, to retrieve the Oura Ring and charger.
