# Supplementary material for: Evaluating the Impact of an App-Delivered Mindfulness Meditation Program to Reduce Stress and Anxiety During Pregnancy: Pilot Longitudinal Study
Source: JMIR Pediatr Parent. 2023 Dec 25;6:e53933. doi: 10.2196/53933 (PMC10775027; doi:10.2196/53933)
Supplement: Multimedia Appendix 2 [file pediatrics_v6i1e53933_app2.pdf]

**Screening Measures**	**Baseline Measures**<br><br>**Consent**<br><br>**Product Education**	**4 Day Baseline HRV**	**Start Headspace 2X/day**<br><br>**Wear OR for month-long trial**	**2 week Measures**<br><br>**Mid-study Incentive**	**End of Study Measures**<br><br>**Collection of Oura Ring & Charger**<br><br>**Post-study Incentive**
	Socio-demographic Measure				Post-Assessment Questionnaire
Depression: Edinburgh Depression Scale	Social Support: MSPSS				
Anxiety: GAD-7	Anxiety: GAD-7			Anxiety: GAD-7	Anxiety: GAD-7
	Pregnancy Anxiety: PRAS			Pregnancy Anxiety: PRAS	Pregnancy Anxiety: PRAS
	Stress: PSS				Stress: PSS
